# The Sky

**Published:** 1892-02

**Authors:** 


### THE SKY.

The sky is for all of us. Bright as it is, it is not too bright nor good for human nature's daily food. Sometimes gentle, sometimes capricious, sometimes awful, it is never quite the same for two moments together; almost human in its passions, almost spiritual in its tenderness, almost divine in its infinity, its appeal to what is immortal in us is as distinct as its ministry of chastisement or of blessing to what is mortal.

